# Synthetic microbial consortia based on quorum-sensing for disease therapy

**DOI:** 10.1186/s40643-025-00962-w

**Published:** 2025-11-03

**Authors:** Yufei Guo, Mengxue Gao, Ling Jiang, He Huang, Guangbo Kang, Haoran Yu

**Affiliations:** 1 Institute of Bioengineering, College of Chemical and Biological Engineering, Zhejiang University, Hangzhou, 310058 Zhejiang China; 2 ZJU-Hangzhou Global Scientific and Technological Innovation Centre, Hangzhou, 311200 Zhejiang China; 3 School of Synthetic Biology and Biomanufacturing, State Key Laboratory of Synthetic Biology, Tianjin Key Laboratory of Biological and Pharmaceutical Engineering, Tianjin University, Tianjin, 300350 China

**Keywords:** Synthetic microbial consortia, Engineered bacteria, Quorum sensing, Disease treatment, Live biotherapeutic products

## Abstract

Synthetic microbial consortia (SyMCon), composed of different artificially engineered bacteria, offer a promising alternative to live biotherapeutic products for disease therapy. These microbial communities use a quorum sensing (QS) mechanism that allows for precise and low-interference communication. Compared to current therapy using only one engineered bacterium, they can reduce the metabolic load of one bacterium, thereby increase drug production, and respond to a wider variety of disease-related signals. This review summarizes recent developments and emphasizes the unique advantages of SyMCon, then proposes multiple perspectives of designs for therapeutic SyMCon. Although SyMCon possess advantages in colonization, responding to multiple environmental signals, and delivering high-yield drugs, future developments should focus on orthogonal QS systems, complex genetic circuits, and modular consortia. More complex consortia allow for better therapeutic functionality, and modular consortia allow for the rapid replacement of disease-specific components, which could unlock the potential of the next generation of personalized microbial therapy.

**Graphical abstract:**

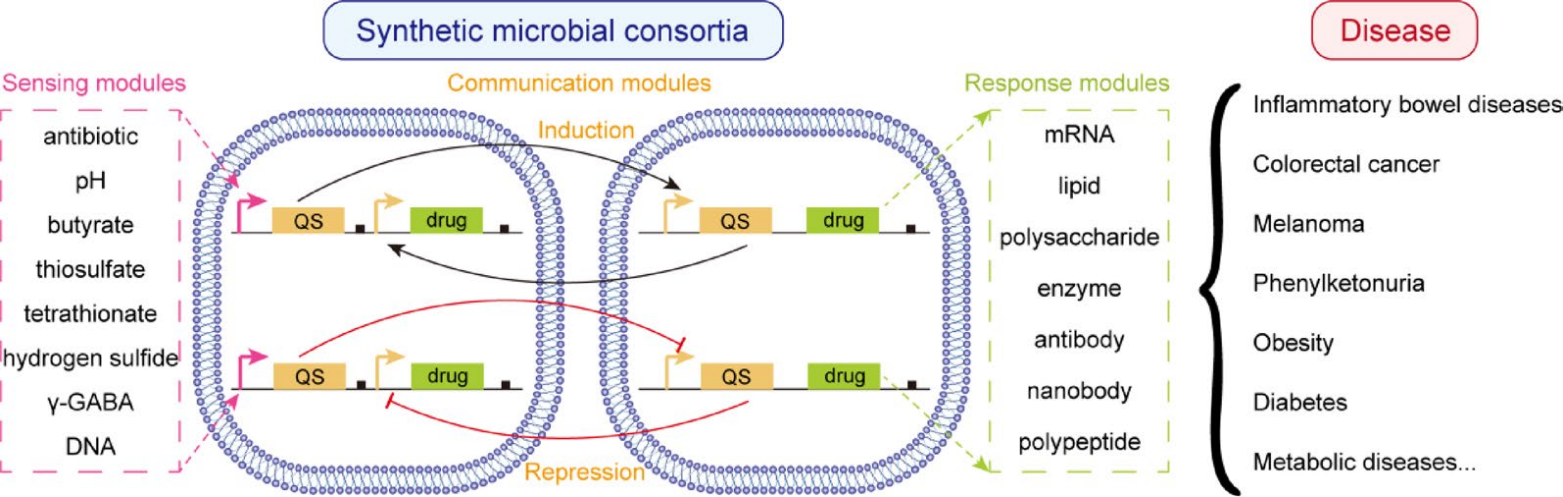

## Introduction

The gut microbiota, often called an important “invisible organ”, plays a significant role in how our bodies function and how our immune system works. When the balance of this microbiota is disrupted, it has been linked to various diseases, including inflammatory bowel disease (Shan et al. 2022), colorectal cancer (Patra et al. 2025), obesity (Puljiz et al. 2023), and diabetes (Paul et al. 2022). Traditional therapies that regulate the gut microbiota, such as probiotics and fecal microbiota transplants, often have limited effectiveness as a result of inconsistent results and a lack of ability to change disease progression (Jayedi et al. 2024). Invasive diagnostic methods like biopsies can delay effective treatment (Zygulska and Pierzchalski 2022). Synthetic biology presents a promising solution by creating synthetic bacteria therapy that can detect harmful signals and then produce treatment molecules on their own. This offers a non-invasive and responsive way to treat diseases (Dang et al. 2023).

However, current methods mostly focus on engineering single strains of bacteria, which has its limitations. A single strain often has a heavy metabolic load (Qian et al. 2017) and struggles to achieve multiple complex tasks including accurate sensing, efficient production and targeted delivery (Qian et al. 2017; McBride and Del Vecchio 2021). To address these challenges, the concept of synthetic microbial communities (SynComs) was proposed, composed of two or more well-defined microbial species (Großkopf and Soyer 2014). SynComs are assembled artificially from natural microbes, have gradually been applied to disease therapy over the past five years (Perez et al. 2021; Jennings and Clavel 2024). With the advancement of synthetic biology, engineered microbes are increasingly used in medical applications. However, single-strain still face challenges such as high metabolic burden and loss of functionality over time. It is necessary to discuss the construction and therapeutic applications of synthetic microbial consortia (SyMCon), composed of engineered microbes. By spreading functions across different strains, SyMCon could lessen the metabolic load, improve production (Sgobba and Wendisch 2020), enhance stability (Li et al. 2024), and allow for coordinated responses to various environmental changes (Grandel et al. 2021). Additionally, they provide strong control mechanisms for timing and location, which is especially important for delivering treatments accurately in the complex gut environment (Wu et al. 2024).

Quorum sensing (QS) is a common method of microbial communication that enables bacteria to cooperate by releasing and detecting signals, such as acyl-homoserine lactones (AHLs), auto-inducer-2 (AI-2) and auto-inducing peptides (AIPs). As a microbial community grows, these signals accumulate. The signals can only induce gene expression when the bacterial density reaches a certain limit (Oliveira et al. 2023). Consequently, the dynamic genetic circuit constructed by QS allows for the precise control of bacterial behavior for density-dependent functions (Song et al. 2014). In SyMCon, QS enables different engineered strains to communicate with each other via signals. This allows SyMCon to work together effectively, mimic ecosystem relationships, and achieve oscillations (McCarty and Ledesma-Amaro 2019).

QS-based SyMCon offer several benefits for synthetic bacterial therapy. First, by distributing tasks among different strains, a single kind of engineered bacterium experiences less metabolic stress and can produce a high yield of therapeutic molecules (Mao et al. 2024). Second, by adjusting the location of the strains, therapeutic efficacy can be improved. One strain can colonize the aerobic intestine to sense signals while another can produce and release therapeutic molecules in the anaerobic tumor (Datla et al. 2017). Third, by combining various functions, SyMCon can implement synergistic therapy. One strain is responsible for improving the metabolic environment while another releases therapeutic molecules. Finally, by adding probiotic consortia, SyMCon can improve the balance of the gut microbiota and enhance the colonization of probiotics (Abisado et al. 2018). For example, compared to the single-strain treatment, synthetic probiotic consortia can enrich vitamin B6 metabolism pathway and alleviate obesity in mice models (Chen et al. 2024a, b).

This review summarizes the advancement in three modules including sensing module, response module and communication module that are necessary for SyMCon. We also discussed the selection of suitable microbial chassis for constructing SyMCon. Furthermore, we propose several design methodologies for SyMCon and describe the applications in disease therapy, which could be useful for developing next-generation intelligent and personalized SyMCon therapy.

## Three essential modules of SyMCon for disease therapy

**Fig. 1:**
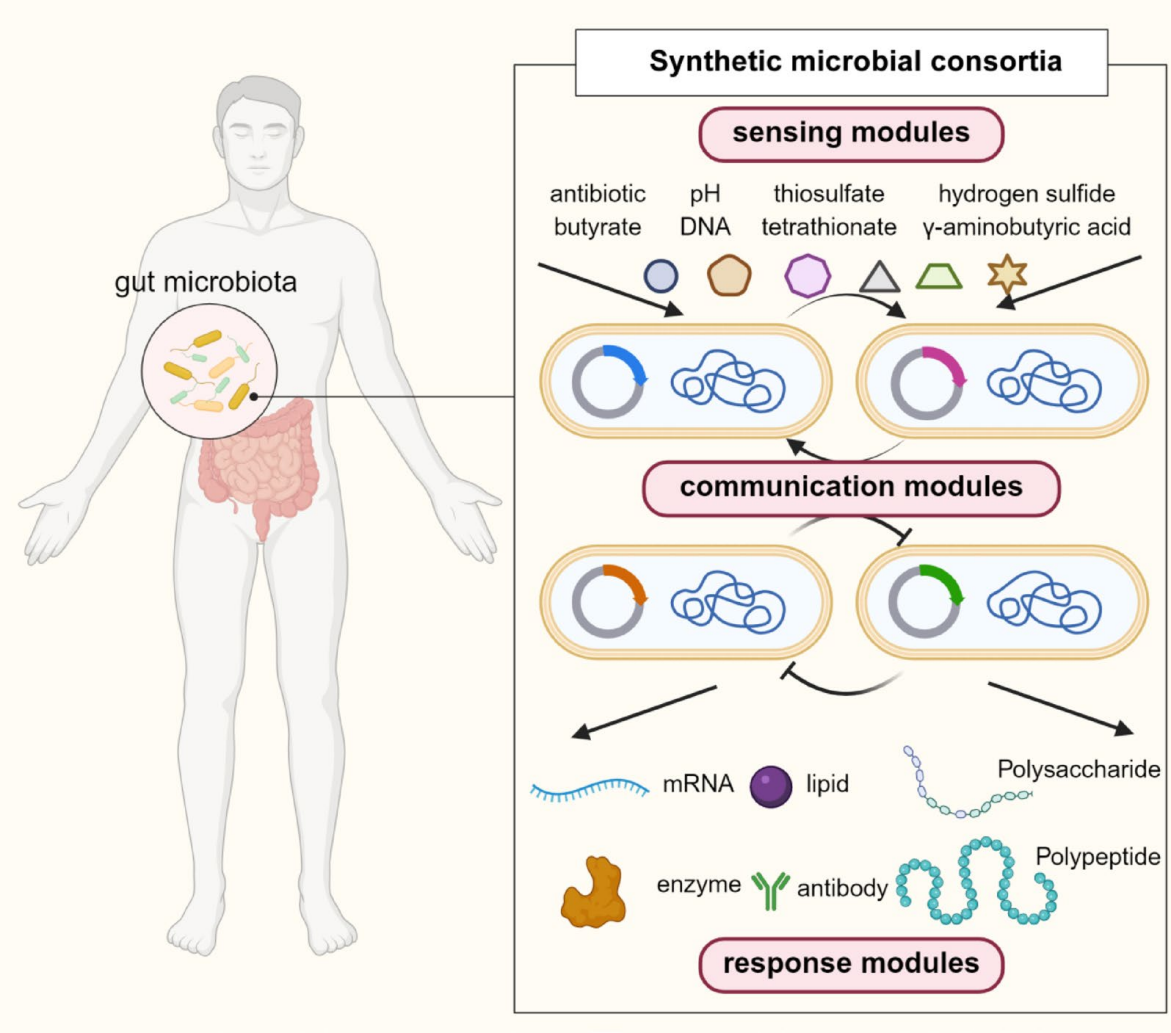
QS-based SyMCon for disease therapy. Sensing modules, communication modules and response modules construct intelligent and versatile microbial consortia

Due to the collaboration of multiple engineered strains, SyMCon have functional diversity compared to single engineered bacteria in therapy (Leeuwen et al. 2023). They can respond to more environmental signals and release more therapeutic molecules. Additionally, special signal systems are necessary for communication. SyMCon operate through three modules: (a) a sensing module that detects pathological signals or environmental stimuli, (b) a response module that produces and releases therapeutic molecules, (c) a communication module that regulates behaviors of different bacteria (Fig. 1). These three modules comprise an integrated framework that demonstrates how QS-based SyMCon can achieve more reliable and effective outcomes than single-strain therapies in clinical settings.

### Sensing modules in engineered bacteria

The rapid development of synthetic biology has established engineered bacteria as promising tools for disease diagnosis and targeted therapy. Through the rational design of biosensors, these microbes can respond precisely to specific environmental cues or disease-associated molecular signals. This enables applications such as tumor detection, monitoring of intestinal inflammation, and management of metabolic diseases. Table 1 illustrates the biosensors in engineered bacteria to sense disease-related small molecules or DNA.

**Table 1:** The summary of sensing modules in engineered bacteria

Disease signals	Disease	Condition	Gene parts	References
Aspirin	Antibiotic therapy	little concentration	*SalR/P*_*sal*_	Chen et al. (2019)
H₂S	Colitis	little concentration	*CpSQR-P*_*L*_*-CstR*	Liu et al. (2019)
Butyrate	Colitis	high concentration	*PpchA-pchA-plEE1*	Bai and Mansell (2020)
NO	Colitis	little concentration	*P*_*norV*_*-NorR*	Chen et al. (2021)
Nitrate	Colitis	little concentration	*NarX-NarL*	Woo et al. (2020)
Thiosulfate	Colitis	little concentration	*ThsS-ThsR-PphsA*	Merk et al. (2024)
Tetrathionate	Human gut epithelium	little concentration	*TtrS-TtrR-P*_*Ttr*_	Zou et al. (2023)
γ-GABA	Colitis	high concentration	*PctC/PhoQ*	Zhao et al. (2024)
Luciferase	Tumor imaging	little concentration	*luxCDABE*	Liu et al. (2021)
KRASG12D mutations	Colon cancer	single-base mutation	*5’homology-KanR-GFP-3’homology*	Cooper et al. (2023)
Oxygen	Colon cancer	anaerobic or microaerobic	*FNRS*	Chowdhury et al. (2019)
			*PpepT*	
			*pVgb*	
			*FF* + *20*	
pH	Colon cancer	acidic	*pCadC*	Chowdhury et al. (2019)
Lactate	Colon cancer	high concentration	*lldR-plldR*	Chowdhury et al. (2019)

A wide variety of environmentally responsive gene circuits have been constructed by modifying natural sensor systems. For instance, an aspirin-inducible circuit was developed using the SalR/Psal regulatory module, allowing *Escherichia coli* Nissle 1917 (EcN) and chromosome-free SimCells to respond to aspirin concentrations ranging from 0.05 to 10 μM, providing a controllable activation platform for oral drug delivery (Chen et al. 2019). Another system utilizes a hydrogen sulfide (H₂S)-mediated QS circuit, in which sulfide/quinone oxidoreductase converts diffusible H₂S into non-diffusible polysulfides, enabling synchronized population-level behavior among engineered bacteria (Liu et al. 2019). A butyrate-responsive biosensor was also constructed by modifying the natural pchA promoter (PpchA-pchA) and the plEE1 element in EcN. This sensor, which uses green fluorescent protein as a reporter, was used to screen for high butyrate-producing bacteria (Bai and Mansell 2020). Additionally, a nitric oxide (NO)-responsive system was developed based on the PnorV promoter and NorR regulatory protein. Incorporation of positive feedback enhanced detection sensitivity to 1.3 nM, and *in vivo* performance was validated in a colitis mice model (Chen et al. 2021).

The integration of multiple biosensors has substantially improved the specificity and precision of engineered bacterial responses. For example, a dual-input AND gate was developed by combining a nitrate-responsive NarX-NarL sensor with a thiosulfate-responsive ThsS-ThsR sensor, enhancing the accuracy of colitis diagnosis (Woo et al. 2020). Another study constructed an AND logic circuit in EcN, enabling the secretion of the anti-inflammatory cytokine IL-22 only when both tetrathionate and anhydrotetracycline (aTc) were simultaneously present. This system demonstrated therapeutic efficacy in germ-free transwell model of the human gut epithelium (Merk et al. 2024). The i-ROBOT system integrates thiosulfate sensing, single-base editing, and therapeutic release into a unified platform. This engineered strain introduces a permanent single-nucleotide mutation into the lacZ gene in response to inflammation. When isolated from fecal samples and plated on X-gal, the resulting blue color intensity serves as a proxy for inflammation severity. Concurrently, the system enables on-demand secretion of the immunomodulatory protein AvCystatin, effectively alleviating symptoms in DSS-induced colitis mice models (Zou et al. 2023).

High-specificity biosensors have also been developed for neurotransmitter. A recent platform based on the PctC/PhoQ system allowed selective detection of γ-aminobutyric acid (γ-GABA), and was used to screen high-producing *Corynebacterium glutamicum* strains via microfluidic droplet sorting (Zhao et al. 2024).

Engineered bacteria have also been adapted for DNA mutation detection using natural competence and horizontal gene transfer pathways. A modified *Acinetobacter baylyi* strain employing a CRISPR-based system successfully detected KRASG12D oncogenic mutations in colon cancer DNA with single-base resolution, and the design is generalizable for other pathogenic variants (Cooper et al. 2023).

Beyond biosensors, the intrinsic tumor-targeting property of EcN enables non-invasive diagnosis and imaging. Several studies have utilized EcN expressing luxCDABE luciferase to detect tumor localization *in vivo* (Liu et al. 2021). Additionally, a synthetic strain capable of producing salicylic acid significantly elevated urinary salicylate levels in tumor-bearing mice, demonstrating the feasibility of environmental signal amplification for non-invasive cancer diagnostics (Gurbatri et al. 2024).

These advances highlight the growing capacity of engineered bacteria to sense diverse disease-related signals. Although current studies focus on synthetic single-strain, these biosensors can be used in SyMCon with complex gene circuits. Consequently, it can achieve sophisticated and spatiotemporally coordinated diagnostics for future applications in precise and personalized therapy.

### Response modules in engineered bacteria

Advances in synthetic biology have significantly expanded the therapeutic potential of engineered bacteria, enabling the expression of functional proteins for disease intervention. Through the integration of genetic circuits, metabolic pathway reprogramming, and smart control modules, engineered bacterial platforms have been developed for targeted drug delivery, immune activation, and tumor suppression. Table 2 summarizes the molecules which can be delivered by therapeutic SyMCon.

**Table 2:** The summary of response modules in engineered bacteria

Medicine	Disease	Gene parts	Administration	References
Phenylalanine ammonia lyase	Phenylketonuria	*PAL*	Oral	Isabella et al. (2018)
L-arginine repressor gene	Colon cancer	*ArgR*	Intravenous injection	Canale et al. (2021)
3-hydroxybutyrate synthase	Colitis	*PhaA-PhaB-TesB*	Oral	Yan et al. (2021)
Flt3L and OX40 ligand	Colon cancer	*Flt3L-OX40L*	Intratumoral injection	Zhu et al. (2022)
CD47 nanobody, PD-L1 nanobody	Colon cancer	*CD47nb, PDL1nb*	Intratumoral injection	Chowdhury et al. (2019)
CDA synthase	Melanoma	*dacA*	Intratumoral injection	Leventhal et al. (2020)
TNF nanobody	Colitis	*TNF Nb*	Oral	Lynch et al. (2023)
Packaging protein	Melanoma	*gD*	Subcutaneous injection	Ma et al. (2024)
β-glucan	Melanoma	*BG*	Subcutaneous injection	Chen et al. 2024a, b)
Insulin	Diabetes	*Insulin-TEVcs*	Oral	Wang et al. (2024a, b, c)
Azurin	Melanoma	*Azurin*	Intravenous injection	Gao et al. (2024)
Cytotoxic hemolysin E	Colon cancer	*HlyE*	Oral	Zhou et al. (2024)
ΦX174E	Colon cancer	*ΦX174E*	Intratumoral injection	Din et al. (2016)

Engineered strains can be programmed to secrete enzymes that degrade toxic metabolites or synthesize therapeutic compounds to restore metabolic balance. One clinical-stage example is SYNB1618, an EcN strain expressing phenylalanine ammonia lyase. Oral administration of this engineered bacterium significantly reduced blood phenylalanine levels in both phenylketonuria mouse models and non-human primates, with clinical trials progressing to Phase II (Isabella et al. 2018). Another engineered EcN strain was designed to convert ammonia, a tumor-associated metabolic waste product, into L-arginine. This metabolic reprogramming increased intratumoral arginine concentrations, promoted T cell infiltration, and enhanced the efficacy of PD-L1 blockade, leading to marked tumor volume reduction (Canale et al. 2021). Similarly, a high-yielding 3-hydroxybutyrate-producing EcN strain has been constructed and shown to reduce inflammatory cytokines IL-6 and TNF-α in a DSS-induced colitis model. The strain also promoted the colonization of beneficial *Akkermansia* spp. and maintained stable persistence in the gut about 14 days (Yan et al. 2021).

Engineered bacteria have also been utilized for local delivery of immune-modulating proteins, including antibodies and antigens, to reshape the tumor microenvironment. For instance, a recombinant *Lactococcus lactis* strain secreting a fusion protein of Flt3L and OX40L was shown to activate natural killer cells and cytotoxic T lymphocytes within tumors. This strategy converted immunologically “cold” tumors into “hot” ones and reversed resistance to anti-PD-1 therapy (Zhu et al. 2022). A synchronized lysis circuit (SLC)-enabled EcN strain has been developed to periodically release CD47 nanobody and PD-L1 nanobody when EcN sense the hypoxic, acid and high-lactate signals in tumor microenvironment. This approach improved survival in a murine lymphoma mice model and induced regression of distant, untreated tumors, suggesting the establishment of systemic anti-tumor immunity (Chowdhury et al. 2019). Another therapeutic strategy employed EcN engineered to express a cyclic di-AMP synthetase for local production of the STING agonist CDA. This strain enhanced antigen presentation and inhibited tumor growth across multiple mouse models while promoting long-term immune memory (Leventhal et al. 2020). In the context of inflammatory bowel disease, an engineered PROT3EcT system was constructed using a modified type III secretion system to secrete anti-TNF-α nanobody. A single prophylactic dose in a DSS-induced colitis mice model significantly reduced inflammatory markers and histopathological damage (Lynch et al. 2023). Photo-controllable therapeutic platforms have also been developed. A light-inducible delivery system (ΔTrim-TPD) enabled specific degradation of HSV-1 glycoprotein gD by releasing anti-capsid protein antibodies, suppressing viral replication. The same system was adapted to target the oncogenic transcription factor c-Myc in a murine melanoma model, demonstrating therapeutic versatility (Ma et al. 2024). A recently developed vaccine platform integrated tumor antigens and β-glucan into a probiotic strain, inducing a trained innate immune response. This approach increased the proportion of M1-like monocytes and reduced recurrence in postoperative tumor treatment (Chen et al. 2024a, b).

Moreover, the delivery systems have further enhanced the therapeutic precision of engineered bacteria by enabling localized drug release while minimizing off-target toxicity. For example, a pH-sensitive EcN robot was engineered to release doxorubicin drug—a DNA and RNA synthesis inhibitor. This system improved intratumoral drug penetration and significantly increased the area of necrosis in tumor derived from HCT-116 cells (Wang et al. 2023). A PASS system was developed to enable rapid insulin release in diabetic mouse models. Light-controlled protease activation triggered insulin secretion, achieving normoglycemia. Compared to traditional transcriptional regulation systems, this response was faster (Wang et al. 2024a, b, c). An ultrasound-inducible circuit was also constructed by combining a heat-sensitive promoter with therapeutic payloads such as the anti-cancer protein azurin and a PD-L1 nanobody. In a melanoma mice model, this system reduced tumor burden while minimizing systemic toxicity compared to conventional chemotherapy (Gao et al. 2024). Another strategy utilized XOR logic gate to amplify tumor-specific signals. An engineered bacterium was programmed to release the cytotoxic hemolysin E (HlyE) only when a tumor-specific input signal was detected. In an AOM/DSS-induced colorectal cancer mice model, this approach reduced polyp formation while preserving the abundance of beneficial *Lactobacillaceae* strains such as NK4A136 (Zhou et al. 2024).

In cancer immunotherapy, QS circuits have been employed to achieve spatiotemporal control over the release of immune checkpoint inhibitors, thereby enhancing therapeutic efficacy. A SLC based on the LuxI/LuxR system was developed to regulate bacterial population dynamics and enable drug release. As bacterial density increases, the LuxI-generated signal accumulates to a threshold, activating the pLux promoter and triggering expression of the bacterial lysis protein ΦX174E, which in turn causes cell lysis and releases anti-CD47 nanobody at the tumor site (Din et al. 2016). This approach was further refined by engineering EcN to stably express and release PD-L1 nanobody and CTLA-4 nanobody. In low-immunogenic tumor models, the system elevated intratumoral levels of granulocyte–macrophage colony-stimulating factor (GM-CSF), promoted tumor regression, and induced systemic antigen-specific immune responses that suppressed the growth of distant, untreated tumors (Gurbatri et al. 2020). Additional designs incorporated attenuated *Salmonella* strains engineered with LuxI-responsive circuits. Upon reaching a certain bacterial density in tumors, these strains sequentially expressed sfGFP for real-time imaging, HlyE for cytolysis of tumor cells, and lysis proteins for HlyE release. The circuit exhibited robust 3-h oscillations in microfluidic systems and demonstrated antitumor efficacy in a liver-metastatic colorectal cancer mice model.

### Communication modules based on quorum sensing

#### Quorum sensing modules and their orthogonality

In Gram-negative bacteria, QS primarily relies on AHLs, such as LuxI/LuxR, LasI/LasR and TraI/TraR. At first, LuxI are synthesized continuously, diffuse into the extracellular environment. When microbial consortia reach a certain density, LuxI is sensed by LuxR-type receptors, either in the same or different bacterial species. At the intraspecies level, in *Pseudomonas aeruginosa*, the LasI/LasR system controls the production of 3-oxo-C12-HSL, which regulates biofilm formation and the expression of virulence factors in high density (Zahir et al. 2024). At the interspecies level, AHL-mediated signals modulate cooperative behaviors among diverse microbial species, thereby enhancing adaptation and survival. In addition, AHLs can modulate microbial metabolic functions, such as upregulating genes involved in microcystin degradation, thereby increasing biodegradation efficiency (Zhou et al. 2025).

In Gram-positive bacteria, QS primarily relies on AI-2 and AIPs. AI-2, synthesized by LuxS, enables interspecies communication via LuxP or LsrB receptors. AIP signaling is typically transduced through histidine kinases, such as AgrC, and governs behaviors of microbial conso, including sporulation and virulence expression (Waheed et al. 2020). For example, *Staphylococcus aureus* utilizes AIP signals to regulate function about pathogenicity.

To prevent signal crosstalk and enable modularity in SyMCon, orthogonal QS systems have been designed. LasI/LasR and TraI/TraR system, LuxI/LuxR and EsaI/EsaR system exhibit orthogonal signals communication. AI-2 signaling has been utilized to mediate interactions between *Escherichia coli* (*E. coli)* and *Salmonella*, offering a foundation for orthogonal communication networks in SyMCon. This ensures independent signal communication in multispecies co-cultures and supports the integration of interspecies communication for collaboration (Wang et al. 2022a, b). By enhancing the concentration thresholds of AHLs, microbial community resilience can be increased to regulate environment stress. By reducing the concentration thresholds of AHLs, the growth dominance of specific strains can be suppressed, promoting community diversity and ecological stability (Gao et al. 2025).

Gene circuit design with orthogonal QS is a fundamental strategy for constructing and regulating SyMCon. QS-based SyMCon enable low-interference communication, adjustable signal intensity, which maintains microbiota balance and improves therapeutic efficacy. Additionally, SyMCon can use the density-dependent QS mechanism to produce toxic proteins at high bacterial density, which causes programmed death to enhance biosafety. These features are valuable in medical applications, as they allow SyMCon to release therapeutic molecules precisely at disease sites while reducing systemic side effects.

#### QS modules in synthetic single-strain

Recent advances in QS-based engineering bacteria have made it possible for single bacterial systems to control the release of therapeutic molecules, autonomously in response to bacterial density (Moon et al. 2012). These designs incorporate QS-regulated gene circuits that coordinate protein production, lysis, and signal-responsive feedback. This offers precise dosing strategies for treating tumors, inflammation, and other diseases.

An QS-based circuit has been constructed using the EsaI/EsaR system. In this circuit, the accumulation of the EsaI signal molecule activates the P_esaS_ promoter while repressing the P_esaR_ promoter. Combining this circuit with protease-regulated elements forms a population-level oscillator that maintains rhythmic activity for up to 72 h under non-microfluidic conditions. This enables sustained, temporally controlled therapeutic delivery (Gu et al. 2023).

Studies have shown that serum albumin can bind to 3OC12-HSL, thereby inhibiting QS signals in *Pseudomonas aeruginosa*. This finding suggests a potential strategy for reducing the virulence of gut pathogens by interfering with multiple QS pathways (Mukherjee and Bassler 2019). A multilayered gene circuit was developed by integrating the LuxI/LuxR system with the AraC (arabinose-inducible), LacI (lactose-responsive), and tetR (tetracycline-responsive) promoters. This design combines two two-input AND gates into a four-input logic gate, allowing for the creation of intricate genetic circuits within a single bacterial strain (Cui et al. 2021).

A dynamic, QS-regulated feedback system has also been designed, which alternates between growth and production modes. During cell proliferation, a LuxI-regulated positive feedback loop activates the TCA cycle. When growth ceases, a LuxR-regulated negative feedback loop inhibits the TCA cycle and initiates acetate synthesis. This approach increases acetate yield and enables stable production in *E. coli* cultures (Soma et al. 2021).

These studies demonstrate that precise, dynamic regulation of metabolic pathways in engineered bacteria can be achieved using modular and orthogonal genetic circuits. However, the metabolic burden associated with single-strain systems is still a significant obstacle to developing multifunctional therapeutics. This challenge underscores the necessity of cooperative, multi-strain consortia that can distribute functional tasks among different microbial members. In this context, quorum-sensing provides the essential communication framework that enables such cooperative consortia to coordinate therapeutic functions effectively within the host.

#### QS communication modules in synthetic microbial consortia

Although single-strain QS systems provide a certain degree of control, multi-strain SyMCon could enable spatially distributed cooperative QS regulation. QS-based genetic circuits form the foundation of SyMCon design, allowing for the modular regulation of interspecies communication, therapeutic specificity, and spatiotemporal precision in complex environments (Alnahhas et al. 2019). These systems use natural signaling and orthogonal regulatory elements to coordinate population behavior while minimizing unwanted communication with the host or endogenous microbiota.

**Fig. 2:**
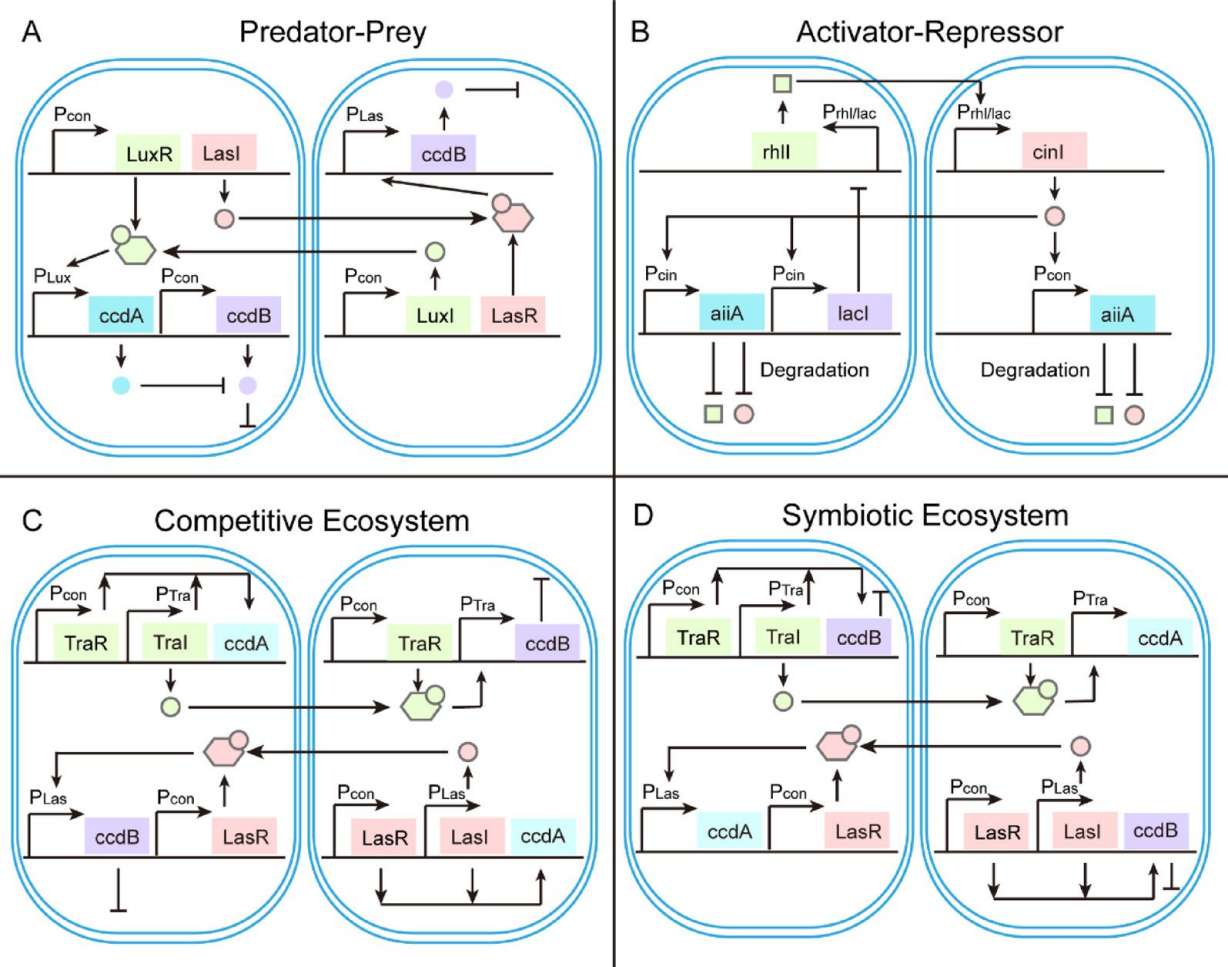
SyMCon consist of two strains which communicate with QS signals. **A** Predator–Prey system. The left “predator” strain triggers toxin expression in the right “prey” strain. This reduces the prey population, which limits predator growth. As the prey population slowly recovers, antidote production enables the predator population to revive. **B** Activator-Repressor system. The left “activator” strain and the right “repressor” strain communicate via rhl and cin systems. And both strains can produce the aiiA enzyme, which degrades both signaling molecules. This results in another layer of negative feedback. **C** Competitive ecosystem. This microbial consortium can mutually inhibit growth. The expression of ccdB in each strain was induced by the HSL produced by the other strains. The left strain produced a small amount of green 3OC6-HSL signal at low population density. Once sufficient signals had accumulated, ccdA and ccdB were activated in both strains. Concurrently, the right strain produced red 3OC12-HSL signal, which resulted in ccdA expression in the right strain and ccdB expression in the left strain. **D** Symbiotic ecosystem. This microbial consortium can mutually protect each growth. Expression of ccdA in each strain was induced by HSL produced by the other strain. The left strain produced green 3OC6-HSL signal to activate ccdB expression in the left strain and ccdA expression in the right strain. Concurrently, the right strain produced red 3OC12-HSL signal, resulting in ccdB expression in the right strain and ccdA expression in the left strain

On the one hand, QS networks facilitate sophisticated coordination strategies, such as unidirectional signal and bidirectional feedback, through cooperation and division of function across strains. In the gut, endogenous microbial consortia use AI-2 molecules to regulate interspecies balance (Liu et al. 2022). In addition, AI-2 signals can repair intestinal damage and reduce inflammation (Sun et al. 2024). Moreover, Synthetic bacteria can similarly deploy diverse signals to restore microbial composition following antibiotic-induced dysbiosis and suppress colonization by pathogens, such as *Staphylococcus and Klebsiella* species (Jennings and Clavel 2024). One striking application of QS is in oscillatory population dynamics to enable periodic therapeutic interventions (Fig. 2A) (Balagaddé et al. 2008). A canonical example is the predator–prey system engineered in *E. coli*, where QS mediates reciprocal regulation between two strains. A number of “predator” bacteria secrete LasI signals that trigger toxin production in the “prey” bacteria, reducing “prey” population. A few “prey” bacteria express few LuxI signals, thereby reducing antidote production and limiting predator growth predator growth. As the “prey” slowly recovers, antidote production enables the revival of the predator. Then, a high-density predator can reduce the prey again. This system can result in cyclic oscillations and steady-state coexistence. The “activator and repressor” system can enable oscillating gene circuits (Fig. 2B). However, it uses a different signaling molecule and supplements the degradation pathway of that molecule for more precise regulation (Chen et al. 2015). Synthetic competitive and symbiotic ecosystems were reconfigured between communication and effector modules. In this system, two completely orthogonal Tra and Las QS systems are selected for communication, while the toxin-antitoxin system is selected as an effector module. The mutual growth inhibition between the two strains is achieved by separately inducing the expression of toxin ccdB in the other strain by signaling molecules produced by the two strains (Fig. 2C and D) (Jiang et al. 2022). Furthermore, nutrients and toxin dynamics can also influence the behavior. The few nutrients and high rates of toxin diffusion could suppress cyclic oscillations (Kuhn et al. 2023).

**Fig. 3:**
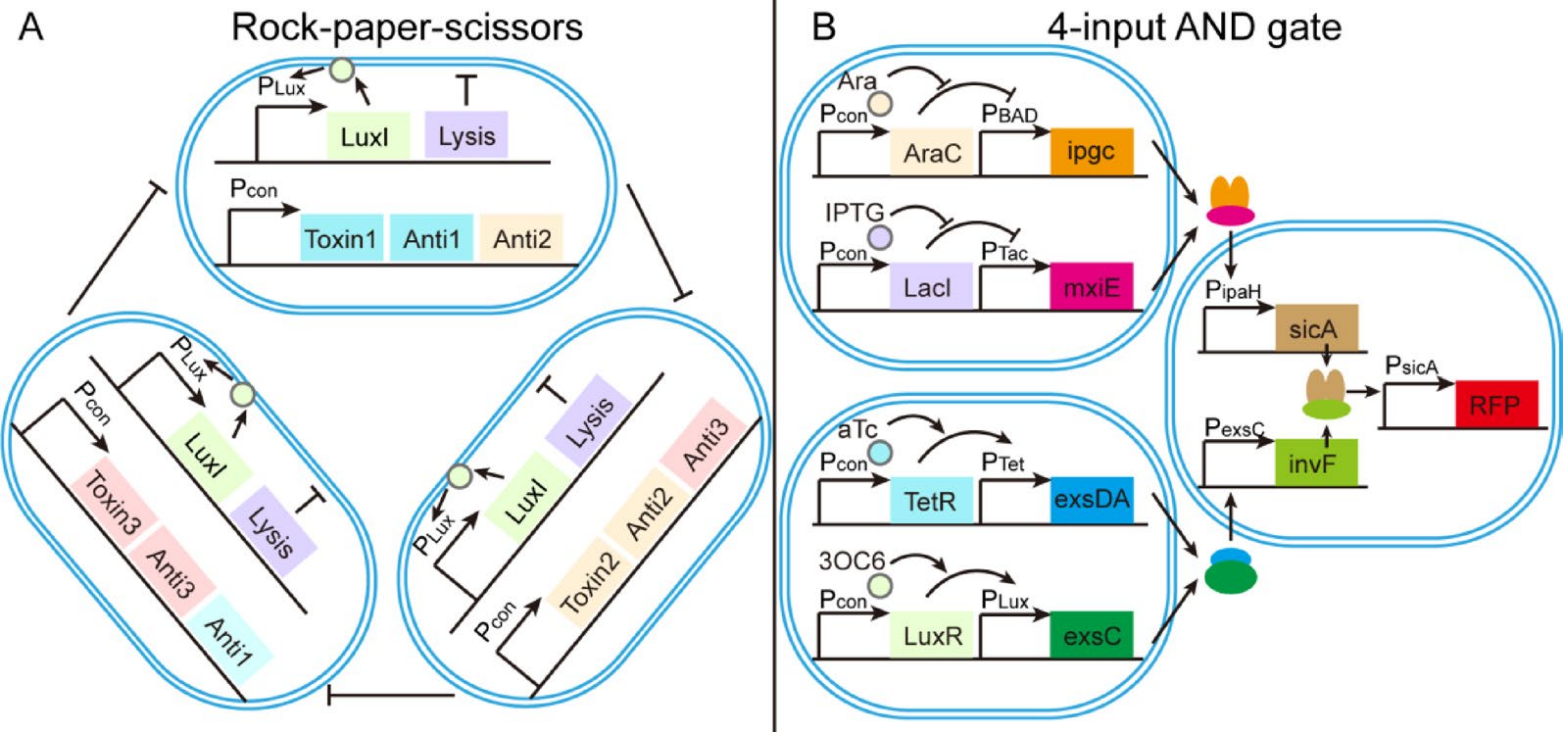
SyMCon consist of three strains. **A** Rock-paper-scissors system. Each strain could kill or be killed by one of the other two strains. **B** 4-input 1-output AND system. Input signals and promoters are arabinose (P_BAD_), IPTG (P_TAC_), aTc (P_Tet_) and 3OC6-HSL(P_Lux_). Each signal was transcriptionally fused to AND gates based on either mxiE-ipgC or exsDA-exsC. Then, two of these gates induced the expression of InvF-SicA*. The output red fluorescent protein (RFP) was on only when all of the inducers were present

On the other hand, the “rock-paper-scissors” circuit stabilizes a microbial consortium of three strains via cyclic inhibition. In a three-strain *E. coli* system using the LuxI/LuxR module, each strain inhibits another while being inhibited in return, promoting population turnover and eliminating genetically unstable mutants. This SLC increases genetic robustness by minimizing circuit loss through plasmid degradation or mutation (Fig. 3A) (Liao et al. 2019). Then researchers optimize “rock-paper-scissors” system through (a) the inhibition of protein production, (b) the digestion of genomic DNA, and (c) the disruption of the cell membrane (Liao et al. 2020). Another innovative design is 4 input AND gate. Three 2 input AND gates were connected together to create a 4 input AND gate in the bacteria. The input signals are Ara (arabinose), IPTG (isopropyl β-D-1-thiogalactopyranoside), 3OC6 (N-(β-ketocaproyl)-L-homoserine lactone), and aTc (anhydrotetracycline). Ara and IPTG bind to the inhibitors AraC and LacI, and the inhibitors can activate transcription of promoters P_BAD_ and P_Tac_. 3OC6 and aTc bind to regulators LuxR and TetR, then they activate transcription of promoters P_Lux_ and P_Tet_. Ara and IPTG constitute an AND gate, and 3OC6 and aTc constitute an AND gate. The two outputs constitute the third AND gate (Fig. 3B) (Moon et al. 2012).

In addition, QS-based SyMCon excel at distributing metabolic workloads, which reduces the burden on individual strains and enhances biosynthetic output. A notable example is the co-culture production of salidroside using two engineered *E. coli* strains programmed with orthogonal QS gene circuits: The first is the QS-SLC (quorum sensing-synchronized lysis circuit), and the second is the QS-MTS (quorum sensing-metabolic toggle switch) (Wu et al. 2021). AG cells metabolize xylose and undergo QS-triggered lysis at threshold density. This releases β-glucosidase, which converts cellobiose into glucose. GD cells then use this glucose to drive the TCA cycle and, once they reach quorum, redirect metabolic flux toward salidroside synthesis by suppressing the activity of citrate synthase CitA. This architecture demonstrates how QS coordination enables the production of metabolites in a resource-efficient and dynamic manner.

Moreover, to address the complexity of SyMCon design and facilitate translational scalability, computational strategies have emerged as indispensable tools (Quinn-Bohmann et al. 2025; Wang et al. 2025). Artificial intelligence (AI) offers predictive modeling of inter-species interactions and optimization of genetic circuit. However, its accuracy remains limited by the scarcity of high-quality and specific microbial interaction datasets. Key approaches include machine learning on multi-omics datasets, generative models for synthetic gene circuits, and reinforcement learning for dynamic behavior control. Several tools have been developed to streamline bottom-up SyMCon engineering. MiMiC enables data-driven selection of minimal microbial consortia from shotgun metagenomic data, preserving essential functional traits while simplifying system complexity (Kumar et al. 2021). AutoCD automates the design of consortia with nutrient competition and QS-mediated bacteriocin regulation to maintain ecological balance (Karkaria et al. 2021). Additionally, the Design–Build–Test–Learn (DBTL) framework integrates molecular circuit construction with community-scale interaction modeling, allowing iterative optimization of both stability and output (Wu et al. 2024). In this framework, orthogonal QS molecules serve as communication modules. Then SyMCon can divide tasks among different microbial members, enabling them to perform complex biosynthesis more efficiently (Zhang and Lyu 2023).

In conclusion, communication modules synchronize the other two functions across multiple strains, ensuring stable colonization and coordinated treatment in complex environments, such as the gut or tumor tissue. Well-designed communication modules based on QS prevent crosstalk and improve the precision of drug delivery, sustaining long-term therapeutic efficacy.

## Selection of natural microbial consortia as chassis strains

Natural microbial consortia have intricate networks and functions, which makes amounts of options for choosing appropriate chassis strains (Cheng et al. 2022). For instance, in the human gut microbiota, *Faecalibacterium* and *Butyrivibrio* work together to break down dietary fibers and help protect against type 2 diabetes (Wang et al. 2024a, b, c). The 14 selected commensal microbes can colonize in intestine and promote intestinal maturation and immunity (Romero et al. 2022). Similarly, *Colwellia* and *Roseovarius* have a symbiotic relationship, where *Colwellia* makes lower ligand α-ribazole of vitamin B_12_, and then *Roseovarius* changes them into active forms, which can increase yield of vitamin B_12_ (Wienhausen et al. 2024).

Biosafety is essential criteria for selecting a chassis strain. The pathogenic bacteria cannot be chosen for disease therapy. *E.coli* can synthesize colibactin, which derives genotoxins (Pleguezuelos-Manzano et al. 2020). And *Fusobacterium nucleatum* have been linked to immunosuppression through TLR/NF-κB signaling (Shi et al. 2024). Consequently, EcN is frequently used in engineered bacterial therapeutics because it lacks virulence factors and has been approved for clinical trials. The expression of its adhesins can promote the effective colonization of the intestinal epithelium (Chen et al. 2023). Other high-potential bacteria include *Bacteroides fragilis* (Roustapoor et al. 2025), *Lactobacillus* spp. (Yang et al. 2025a, b), and *Lacticaseibacillus rhamnosus* GG (LGG) (Leser and Baker 2024).

Second, microbial consortia can improve colonization efficiency through cooperative interactions and ecological niche sharing in complex environments such as the gastrointestinal tract (Britton and Young 2014). These interactions include nutrient exchange, competitive exclusion of pathogens, and collaborative surface adherence, all of which contribute to create a favorable colonization environment (Flores et al. 2023). For instance, *Lactobacillus rhamnosus* GG promotes mucus production and tight junction formation, indirectly supporting the stability and persistence. Some microbes can deplete bile salts, thereby reducing stress-induced clearance of bacteria (Collins et al. 2023). QS systems can promote colonization by enabling density-dependent behaviors such as biofilm formation, adhesion, and surface motility (Mukherjee and Bassler 2019).

Moreover, since different body parts have different microbial consortia, a tailored consortia is necessary for each site, such as the oral flora and gut microbiota. Dysbiosis—characterized by the loss of beneficial microbes, overgrowth of pathogens, and disrupted metabolite profiles—is a hallmark of various chronic diseases, including inflammatory bowel disease, metabolic syndrome (Yang et al. 2021), and cancer (Tilg et al. 2020). Therefore, different microbial chassis can be considered according to the disease when choosing a chassis. In addition, different types of tumors also have different intratumoral microbiota. Selecting intratumoral bacteria helps microbial consortia survive in areas of low oxygen and function properly under changing conditions (Nejman et al. 2020). The purple photosynthetic bacteria in tumor tissue can diagnose tumors using near-infrared light. This not only helps reach the tumors, but also boosts immune responses and improves survival rates in breast cancer mice models (Goto et al. 2023).

Finally, pinpointing important species and tweaking their ratios can adjust these consortia for better therapeutic results and microbial stability (Escudero-Martinez and Bulgarelli 2023). A method called the “community-function landscape” has been created to link microbial makeup to their functional results. This approach also demonstrates that when species specialize, it lightens the workload on individual strains (Sanchez et al. 2023). Simpler consortia have less complexity and more predictable behavior, which provides a stable foundation for designing effective microbial consortia (Aggarwal et al. 2023). And the “global epistasis” model can predict the function of microbial consortia when several species have been added (Diaz-Colunga et al. 2024). Additionally, non-genetic approaches, such as temperature-controlled systems that enable the long-term co-culture of *E. coli* and *P. putida* for many generations, support the design of interspecies communities (Lee et al. 2025).

In summary, the successful design of a therapeutic microbial community relies on insights derived from natural microbial communities. Selecting the right natural microbial chassis can help develop safe, stable, and highly effective SyMCon therapy.

## Prospective development of SyMCon for disease therapy

### Early applications of SyMCon in disease therapy

**Fig. 4:**
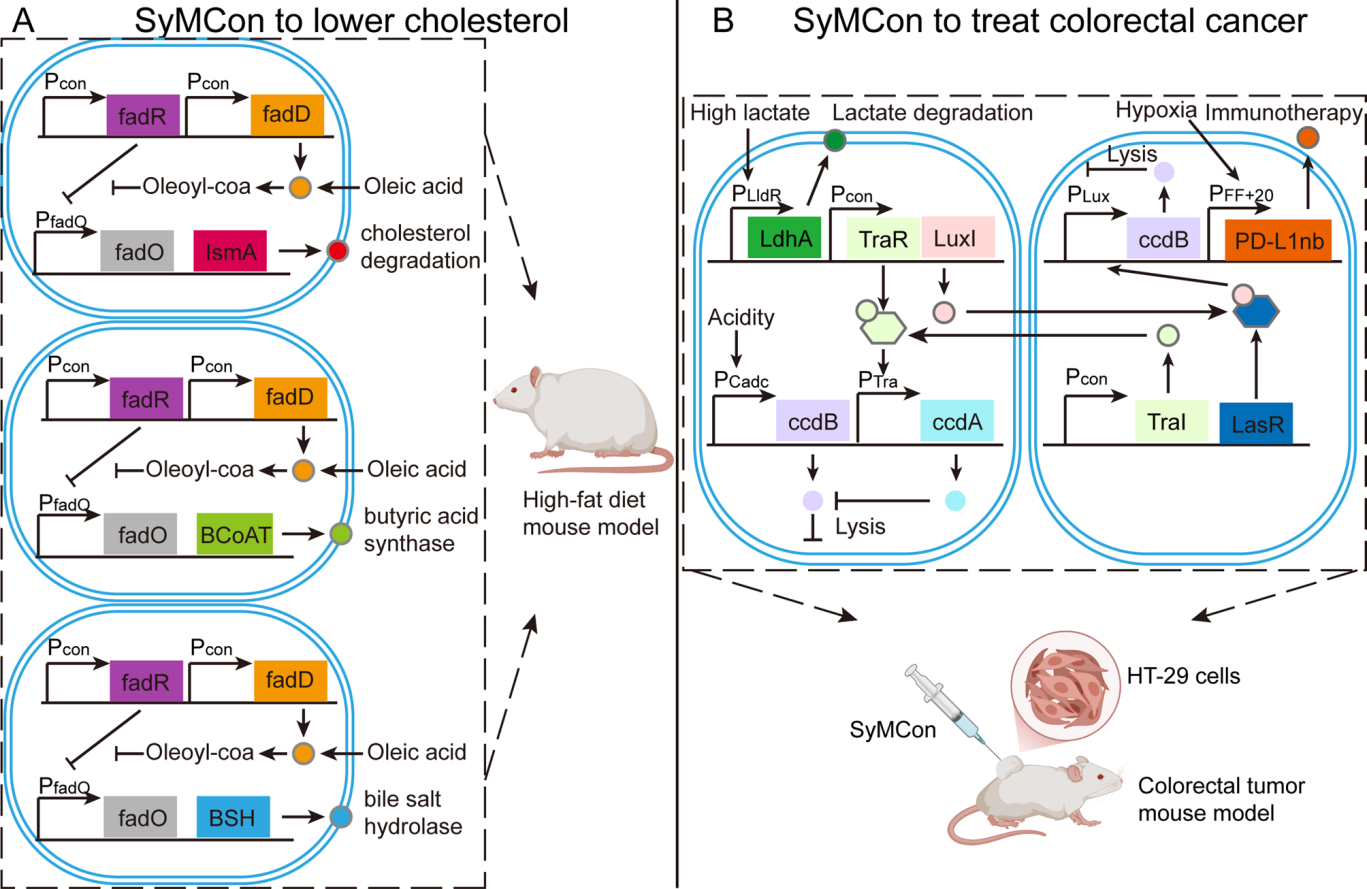
Two early applications of SyMCon. **A** A SyMCon sense oleic acid signal and express the cholesterol degradation enzyme IsmA, butyric acid synthase BCoAT, and bile salt hydrolase BSH, respectively. **B** A SyMCon sense acidity, hypoxia, and high lactate signals of the tumor microenvironment, and express and release lactate dehydrogenase LdhA and anti-PD-L1 nanobody. The two strains can coordinate through orthogonal QS systems

Although SyMCon have been widely applied in industrial fields such as biorefineries, bioremediation, and complex chemical production (Jiang et al. 2023), their exploration in disease therapy is still at an early stage. One example is an oleic acid–inducible SyMCon, composed of three strains engineered to express the IsmA, BCoAT, and BSH genes, respectively (Fig. 4A). This system can degrade cholesterol while simultaneously enhancing butyrate and bile salt hydrolase production. In mice fed a high-fat diet, the consortium was shown to accurately detect lipid levels, lower serum cholesterol, and improve immune function (Yang et al. 2025a, b). Another example is a SyMCon designed to sense three physiological signals of the tumor microenvironment (acidity, hypoxia, and high lactate), and to coordinate the release of lactate dehydrogenase LdhA together with PD-L1 nanobody based on QS systems (Fig. 4B). LdhA helps to degrade intratumoral lactate, thereby enhancing the efficacy of anti–PD-L1 immunotherapy. In mouse models of colorectal cancer, this SyMCon improved the intratumoral metabolic environment and strengthened the efficacy of immunotherapy (Guo et al. 2025). To date, studies on therapeutic SyMCon remain limited, but the field holds broad opportunities for further exploration. Therefore, it is significant to discuss therapeutic SyMCon from the perspectives of gene circuit design, modular strain design, material design, and translational barriers to clinical application. 

### Gene circuit design of SyMCon for efficient and biosafe therapy

**Fig. 5:**
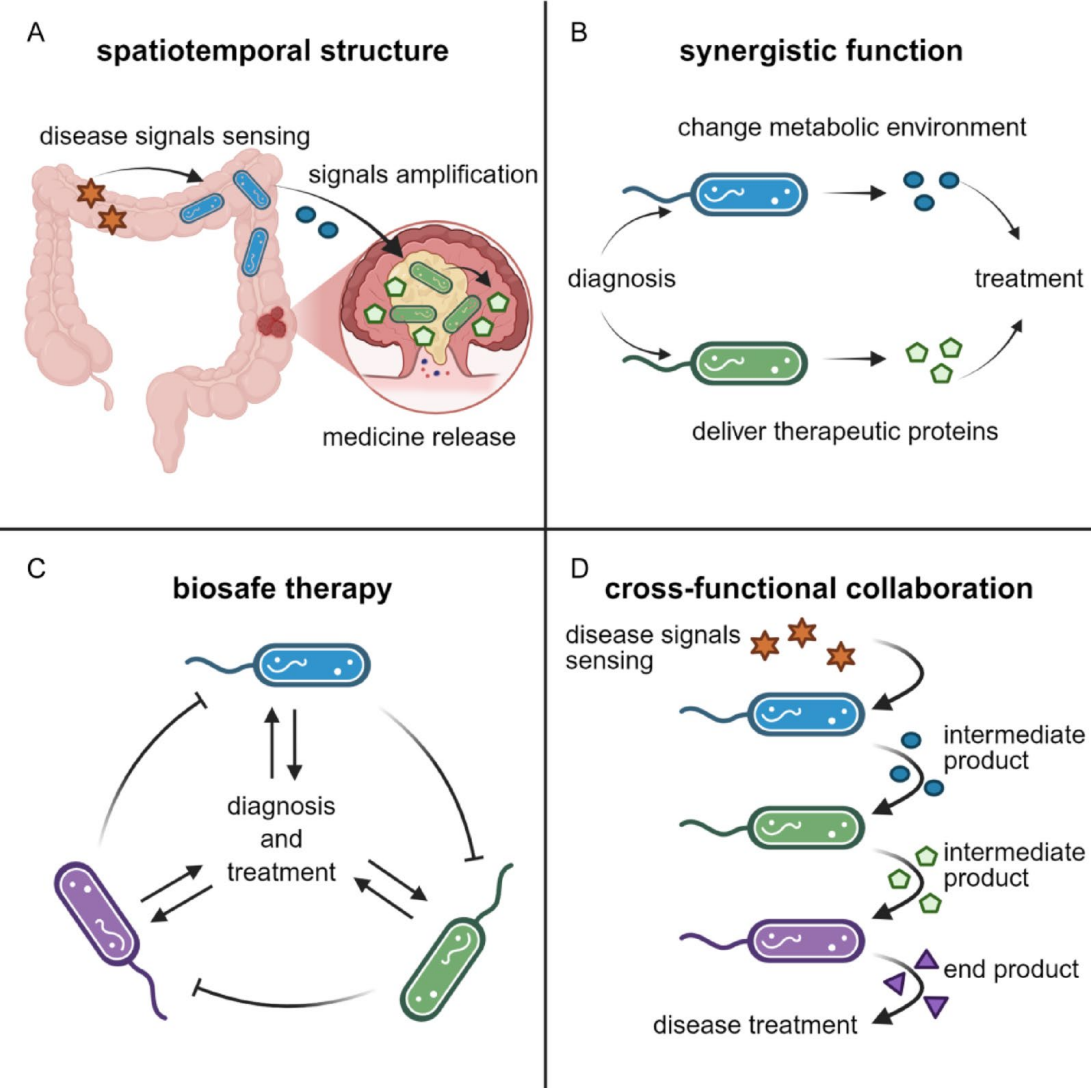
A scheme of genetic design in SyMCon. **A** The division of spatiotemporal structure. **B** The synergistic function to enhance therapeutic efficacy. **C** The biosafe therapy with antagonism of three strains. **D** The cross-functional collaboration to produce therapeutic molecules

Gene circuit design is the key to achieving the multifunctionality of SyMCon, whether the goal is a minimal metabolic burden, high-yield therapeutic molecules, or biosafe microbial communities. The most promising function is spatiotemporal division. In the spatial dimension, colonization of the sensing bacteria and the response bacteria in different body spaces can improve treatment precision and efficiency. For example, one parthenogenetic anaerobic bacterium colonizes the intestines, receives disease signals, and releases QS signals. Another anaerobic bacterium colonizes the anaerobic core region in intestinal tumors. It receives the QS signals and lyses within the tumor to release therapeutic molecules. This layered communication enhances signal specificity and prevents metabolic load in a single bacterium (Fig. 5A). In the temporal dimension, SyMCon can achieve dynamic oscillations, control colony density within a safe range, and prevent functional gene mutation. There are three approaches to design oscillating SyMCon for disease therapy. On the one hand, one strain delivers enzymes that improve the metabolic environment, while the other delivers therapeutic proteins, such as antibodies. These two strains are regulated by QS signals (Fig. 5B). On the other hand, the three strains oscillate synergistically under QS signals regulation to achieve biosafe therapy, preventing continuous reproduction of bacteria (Fig. 5C).

SyMCon also offer a safe solution by integrating gene circuits. Based on QS, they can activate therapeutic gene expression only when the bacterial population reaches a defined density threshold. Because therapeutic gene expression is only triggered when bacterial density exceeds a threshold, this design ensures activation occurs exclusively within the disease microenvironment, avoiding ectopic expression elsewhere. These systems can improve treatment precision while minimizing off-target effects and systemic toxicity. In tumor models, for example, SLC circuits have been employed to control the release of immune checkpoint inhibitors or cytolytic agents when microbial density was achieved, thereby confining drug activity to the tumor site (Liao et al. 2024).

Additionally, as the genetic design in single strain, SyMCon can use Boolean logic circuits to process environmental signal, ensuring that drug release occurs only under defined multi-signal conditions. This strategy improves safety in tissues that may sense several features of the disease microenvironment. Furthermore, spatial control can be achieved by incorporating inducible systems responsive to exogenous physical triggers, such as light or ultrasound. When coupled with endogenous signal detection, these dual-input circuits enable on-demand therapeutic activation with minimal systemic impact (Tegegne and Savidge 2025).

Functional layering can also enhance biosafety. For example, toxin-producing strains can be activated only in the presence of upstream sensing bacteria that detect disease-specific signals. This ensures that cytolytic activity is spatially confined and temporally regulated. Ultimately, by distributing sensing and response modules across a well-coordinated microbial network, SyMCon can achieve sophisticated therapeutic behaviors with high reliability and tunability.

### Modular strain design for personalized therapy

Through rational chassis selection, module selection and genetic circuit design, SyMCon can precisely regulate the behavior of microbial communities and dynamically control therapeutic efficacy in specific disease microenvironments. Through the construction of standardized and modular microbial community pools, SyMCon can achieve rapid assembly of personalized therapy in clinic (Zeng et al. 2023). The modular design of SyMCon allows for quick replacement according to specific patient conditions and quick customization of the most suitable combination of live biotherapeutic products (LBP) (Mehlferber et al. 2024). For example, left-half colon cancer has high expression of epiregulin (EREG) and amphiregulin (AREG) proteins (Lee et al. 2016), while right-half colon cancer has high mutation rates of the TGFbR2 and BRAF genes (Missiaglia et al. 2014). These characteristics can be exploited using sensing bacteria to treat the two types of colon cancer. Besides, the microsatellite instability-high/deficient mismatch repair (MSI-H/dMMR) colon cancers have high PD-L1 expression, and treatment with a PD-L1 antibody is highly effective with low systemic toxicity (Shen et al. 2019). Therefore, responsive bacteria can be used to release PD-L1 antibodies for MSI-H/dMMR colon cancer patients. For patients with weakly expressed PD-L1 colon cancer, bacteria that release highly toxic azurin proteins can be selected.

Three modular strains can produce a natural therapeutic molecule in a step-by-step process, which reduces the metabolic burden on a single strain and leads to higher yields (Fig. 5D). This modular architecture reduces the metabolic burden on individual bacteria, increases stability, and allows for greater functional complexity than single-strain systems can accommodate. This functional segregation allows each strain to be optimized for its specific task. To harmonize the functions of different modular strains, QS mechanism enables communication across multiple strains. A representative example is the co-culture system for salidroside production, in which two *E. coli* strains were engineered with orthogonal QS modules. One strain synthesized β-glucosidase and released it via cell lysis triggered by quorum signals, while the second strain used the glucose to fuel growth and channel carbon flux toward salidroside biosynthesis. This task partitioning enabled dynamic resource sharing and precise control over metabolite flow, thereby improving production efficiency (Wu et al. 2021).

### Material design to minimize competition

When using SyMCon, researchers should consider modulating inoculation ratios, growth rates, and auxotrophic dependency. This can help stabilize the community and prevent the dominance or extinction of strains. Furthermore, increasing community diversity and minimizing niche overlap between engineered strains can reduce inter-strain competition and improve overall colonization (Wang et al. 2024a, b, c). Consequently, to solve the problem of difficult stable coexistence of mixed species microbial communities due to competitive relationships, artificial materials can be used to construct spatial-isolated microbial communities. They have used polymeric microcapsules to keep groups of microbial swarmbots apart, while still allowing the exchange of small molecules. This work achieves more stable communities and better task management in biomanufacturing and consortia assembly (Wang et al. 2022a, b). Moreover, hydrogel- and microgel-based living materials can be coupled with 3D printing to create personalized living materials for maintaining SyMCon (Gao et al. 2023).

### Translational barriers to clinical application of SyMCon

Despite the promising advantages of SyMCon, their translation into medical practice faces several important barriers. Biosafety remains a primary concern. Engineered strains within SyMCon may interact unpredictably with host tissues, produce unintended metabolites, or exhibit off-target effects (Shu and Liu 2025). Ensuring that SyMCon remain safe under diverse physiological conditions will therefore be essential for clinical application. Stability and colonization in the host represent another critical challenge. Compared with single engineered strains, SyMCon are more prone to losing function or balance in complex environments. For example, if one strain fails to sense or respond to its quorum signal, the coordinated activity of the entire consortium may collapse. Similarly, ecological pressures from the host microbiome could disrupt SyMCon ratios and impair therapeutic efficacy. These risks highlight the need to carefully design SyMCon with redundancy, robust signal circuits, and mechanisms to stabilize community composition (Hitch et al. 2025). Patient-to-patient variability in microbiome composition, immune status, and diet further complicates the clinical translation of SyMCon. Individual differences may affect engraftment, persistence, and therapeutic outcomes, raising the need for biomarkers and personalized designs (Liu et al. 2025; Jayaprakash et al. 2025). Finally, regulatory considerations pose another hurdle. At present, there are no established frameworks tailored to multi-strain SyMCon, and existing guidelines are based primarily on single-strain. Developing clear regulatory pathways will be crucial for advancing SyMCon into human trials.

## Conclusion

LBP is a non-invasive and precision treatment for complex diseases, such as obesity, diabetes, and cancer. The rational design of communication provides a platform for the next generation of LBP. This review describes several QS-based SyMCon designs for treating diseases. On the one hand, they can sense and respond to human disease signals, adapting dynamically to the disease microenvironment. Their ability to colonize and release therapeutic molecules at the sites of disease improves therapeutic efficacy and mitigates the toxicity of systemic drug delivery. These LBP has high specificity and minimal burden for human body. On the other hand, by distributing functions among different strains, SyMCon reduce the metabolic burden on a single engineered bacterium. This allows them to achieve greater stability, responsiveness, and therapeutic precision through division of functions and interspecies communication. In addition, AI can address several key issues in SyMCon, including the predictability of interspecific and intraspecific interactions, long-term stability within the host, and constructing modular strains for personalized therapy. Looking forward, SyMCon takes a comprehensive approach to intelligent and personalized disease treatment by integrating microbial ecology, host-microbe interactions, modular design and AI design.

## Data Availability

Not applicable.

## Abbreviations

AHLs: Acyl-homoserine lactones
AI: Artificial intelligence
AIPs: Auto-inducing peptides
AI-2: Auto-inducer-2
AREG: Amphiregulin
aTc: Anhydrotetracycline
CDA: Cytidine deaminase
CD47: Cluster of differentiation 47
CO_2_: Carbon dioxide
DBTL: Design–Build–Test–Learn
*E. coli*: *Escherichia coli*
EcN: *Escherichia coli* Nissle 1917
EREG: Epiregulin
GM-CSF: Granulocyte-macrophage colony-stimulating factor
HlyE: Hemolysin E
H₂S: Hydrogen sulfide
IPTG: Isopropyl β-D-1-thiogalactopyranoside
LBP: Live biotherapeutic products
LGG: *Lacticaseibacillus rhamnosus* GG
dMMR: Deficient mismatch repair
MSI-H: Microsatellite instability-high
MTS: Metabolic toggle switch
NO: Nitric oxide
PD-L1: Programmed cell death-ligand 1
QS: Quorum sensing
sfGFP: Superfolder green fluorescent protein
SLC: Synchronized lysis circuit
SyMCon: Synthetic microbial communities
TCA: Tricarboxylic acid cycle
γ-GABA: γ-Aminobutyric acid
